# Biobased Solvents for Pressurized Liquid Extraction of *Nannochloropsis gaditana* Omega-3 Lipids

**DOI:** 10.3390/md19020107

**Published:** 2021-02-12

**Authors:** Cristina Blanco-Llamero, F. Javier Señoráns

**Affiliations:** Healthy-Lipids Group, Sección Departamental de Ciencias de la Alimentación, Faculty of Sciences, Universidad Autónoma de Madrid, 28049 Madrid, Spain; cristina.blanco@uam.es

**Keywords:** microalgae, alternative green extraction, omega-3 PUFA, *Nannochloropsis*, *Isochrysis*, *Tetraselmis*, renewable solvents, pressurized liquid extraction, ultrasound assisted extraction, glycolipids

## Abstract

To develop greener extraction alternatives for microalgae biomass, ultrasound assisted extraction (UAE) and pressurized liquid extraction (PLE) with different biobased solvents were investigated, demonstrating that both techniques are useful alternatives for algal lipid extraction. Specifically, *Nannochloropsis gaditana* lipids were extracted by UAE and PLE at different temperatures and extraction times with sustainable solvents like 2-Methyltetrahydrofuran (2-MeTHF) and its mixtures with ethanol and other alcohols. The best oil yields for both PLE and UAE of *N. gaditana* were achieved with the mixture of 2-MeTHF:ethanol (1:3), reaching yields of up to 16.3%, for UAE at 50 °C and up to 46.1% for PLE at 120 °C. Lipid composition of the extracts was analyzed by HPLC-ELSD and by GC-MS to determine lipid species and fatty acid profile, respectively. Different fractionation of lipid species was achieved with PLE and solvent mixtures of different polarity. Thus, for the extraction of glycolipids, ethanolic extracts contained higher amounts of glycolipids and EPA, probably due to the higher polarity of the solvent. The optimized method was applied to microalgae *Isochrysis galbana* and *Tetraselmis chuii* showing the potential of mixtures of biobased solvents like 2-methyl-THF and ethanol in different proportions to efficiently extract and fractionate lipids from microalgal biomass.

## 1. Introduction

Microalgae have recently attracted considerable interest as raw materials and have been described as great sources of bioactive lipids as polyunsaturated fatty acids (PUFAs). Microalgae contain polar lipids like glycolipids that include eicosapentaenoic acid (EPA; 20:5 n-3) and docosahexaenoic acid (DHA; 22:6 n-3), and have been widely recognized as bioactive compounds for human health. Indeed, PUFAs produced by microalgae exhibit antioxidant, antibacterial, antiviral, and detoxifying capacities. PUFAs also prevent hypercholesterolemia, improve brain function, and exert good immune stimulatory effects, and have received increasing interest from pharmaceutical and food industries [1,2,3].

On the other hand, *n-3* LC-PUFA dietary supplements are widely available, usually in triacylglycerol (TAG), non-esterified free fatty acid (FFA), and ethyl ester (EE) forms from fish oil, algae, and other sources [4]. Interestingly, it has been shown that EPA and DHA in the form of polar lipids (phospholipids and glycolipids) exhibit better bioactivity and bioavailability than in the TAG or EE forms. DHA polar lipids showed more efficient bioactivity than the TAG, EE, and FFA forms in decreasing hepatic and serum total cholesterol and TAG levels and increasing *n-3* concentration in the brain during a short period. Therefore, the dietary form of PUFA affects their nutritional properties, with polar lipids the ones with the highest importance. However, the natural sources of DHA/EPA bounded to polar lipids are relatively limited compared to those of TAG forms [5,6,7].

*Nannochloropsis gaditana* has been described as a producer of high amounts of valuable lipids. Specifically, it has an important content of triglycerides and polar lipids such as phospholipids and glycolipids. Polar lipids are important structural and functional components of the cell membrane where microalgae have the capacity to accumulate high levels of EPA [8].

In order to explore the potential applications of polar lipids, it is necessary to separate lipids from the biomass in a fast, selective, and green way that does not alter the original algal compounds. Several traditional techniques have been widely used for years for lipid extraction. However, conventional techniques often use toxic solvents and are not efficient nor selective, producing altered or low concentrated extracts, where additional processes such as filtration, fractionation, and concentration would be required [3,9,10,11].

Ultrasound assisted extraction (UAE) and pressurized-liquid extraction (PLE) are valuable extraction alternatives. UAE is a technique that achieves high extraction yields due to a mechanism named cavitation, where bubbles collapse near the sample, causing cell disruption and resulting in a higher release of cell compounds into the solvent [12]. PLE is based on the use of high temperature and pressure to enhance the process and improve extraction yields. The high temperature affects extraction kinetics by increasing mass transfer rates, diffusion and solubility of compounds, and by reducing the viscosity of the solvent, among other effects. Furthermore, since the raw material is retained in the extraction cell, PLE eliminates the need to filter the extract after the process is completed. Additionally, the higher efficiency of the technique allows replacing common organic solvents by environmentally friendly ones. The environmental impact, energy, and time consumed in conventional techniques vs. the alternative ones has also been widely evaluated, showing the advantages and difficulties of UAE and PLE for scale up process and the need to implement these techniques using new green solvents [4,13,14,15].

The aim of this work was to study lipid composition of *N. gaditana* employing advanced extraction techniques such as PLE and UAE and green solvents based on the solvent selection guide of Prat et al. [16], which classified solvents based on their environmental and human health impact. Hence, biobased solvents are derived from renewable sources of biological origin, like agricultural crops rich in carbohydrates. In this work, a new sugarcane or corn biomass derived solvent, 2-methyl-tetrahydrofuran (or 2-MeTHF, CAS No. 96-47-9) was studied as a promising substitute of non-polar conventional solvents such as hexane [17,18], studying mixtures with ethanol, isopropanol, and isobutanol for the advanced extraction of microalgal lipids. As responses to optimize extraction, obtained oil yield, relative percentage of neutral and polar lipids (GL) analyzed by HPLC-ELSD, and omega-3 fatty acids (DHA and EPA) composition in the samples analyzed by GC-MS, were considered. Different lipid species fractionation was achieved with PLE and biobased solvent mixtures of alcohols and ethers of different polarity. The developed methods were applied to other microalgae such as *Isochrysis galbana* and *Tetraselmis chuii*, showing the potential of mixtures of biobased and renewable solvents like 2-methyl-THF and ethanol in different proportions to extract and fractionate lipids from microalgal biomass in a fast and ecological process.

## 2. Results

### 2.1. Ultrasound Assisted Extraction of Nannochloropsis Gaditana

**Figure 1:**
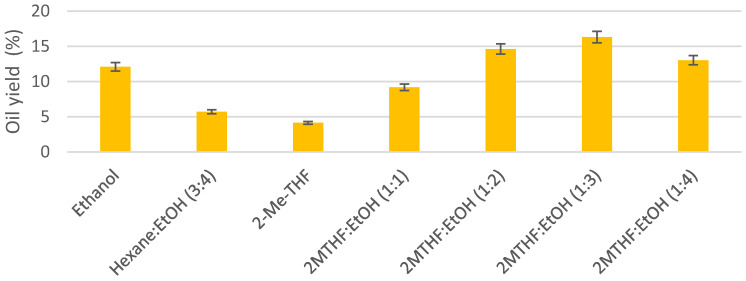
Extraction yields from *N. gaditana* using different solvents at 50 °C and 30 min with ultrasound assisted extraction (UAE) (EtOH: Ethanol, 2MTHF: 2-methyl-THF).

The results of oil yield for UAE from *N. gaditana,* as shown in Figure 1, ranged from 4.13% to 16.32%, depending on the tested solvent, reaching the maximum yield when the mixture of 2-methyl-THF and ethanol (1:3 *v/v*) was used. It is interesting to point out the results with 2-MeTHF only and how the extraction yield increased when it was mixed with ethanol (selected as green and more polar solvent) in any of the tested proportions.

### 2.2. Pressurized Liquid Extraction of Nannochloropsis Gaditana

**Figure 2:**
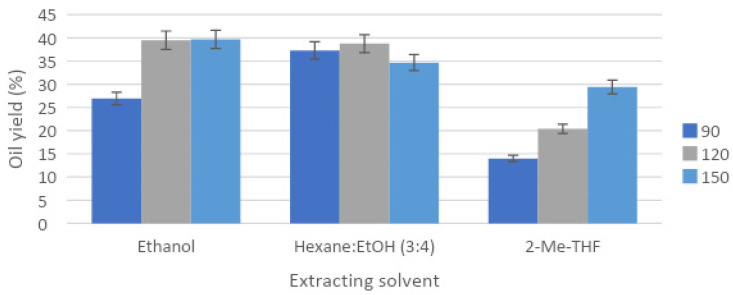
Pressurized-liquid extraction (PLE) yield for *Nannochloropsis gaditana* using different extraction solvents, temperatures (90, 120 and 150 °C) and static extraction time equal to 15 min.

**Figure 3:**
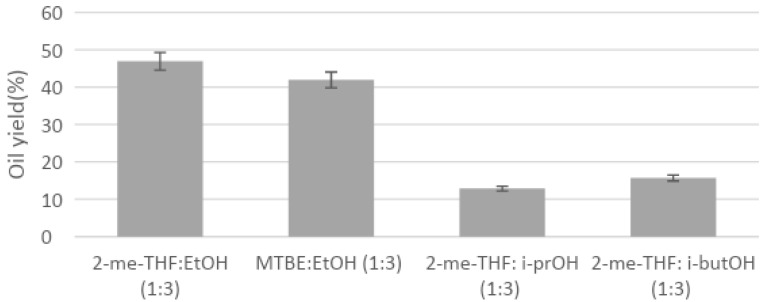
PLE yield for *N. gaditana* using different mixtures of solvents at 120 °C and 15 min of static extraction. 2-MeTHF:ethanol (1:3), MTBE:ethanol (1:3), 2-MeTHF:isopropanol (1:3), and 2-MeTHF:isobutanol (1:3).

In order to compare both PLE and UAE techniques, solvents selected for UAE and the mixture of 2-MeTHF:ethanol (1:3) were employed with PLE in the first place. Additionally, to optimize PLE, mixtures of 2-MeTHF with other less polar alcohols (isopropanol and isobutanol) were tested in the same proportion (1:3), and finally, a mixture of ethanol with another ether of similar properties, methyl-tert-butyl ether (MTBE) was also compared. Extraction yields for different solvents presented in Figure 2 and Figure 3 confirmed that PLE and UAE followed the same trend for algal oil yield: 2-MeTHF produced the lowest yields at every tested temperature in PLE, whereas the mixture of 2-MeTHF with ethanol achieved the best results, followed by only ethanol as the solvent.

Pressurized extraction of *Nannochloropsis* biomass with the different mixtures of ether:ethanol (1:3) showed results that varied greatly from 13.99% to 46.06% (Figure 3), with a large influence of the tested solvent (differences observed at 5% level). In all cases, PLE achieved much higher yields than the ones obtained with UAE (Figure 1), which could be explained because PLE allows reaching much higher temperatures during extraction due to an increase in the system pressure that keeps solvents in liquid state. Higher extraction temperatures are related to the increase in the mass transfer from sample to solvent, involving a higher extraction yield as the temperature increases. Consequently, oil yields increased as the tested temperature did, reaching the highest yield at 150 °C for most of the used solvents.

### 2.3. Analysis of Microalgal Lipids Classes by HPLC-ELSD

**Figure 4:**
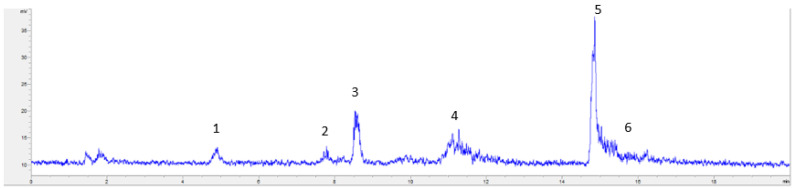
HPLC-ELSD chromatogram of a *N. gaditana* UAE ethanolic extract, shown as an example of *N. gaditana* chemical composition, where peak 1 corresponded to TAG, 2 to FFA, 3 to 1,3-DAG, 4 to 1,2-DAG, 5 to GL, and 6 to MAG.

**Figure 5:**
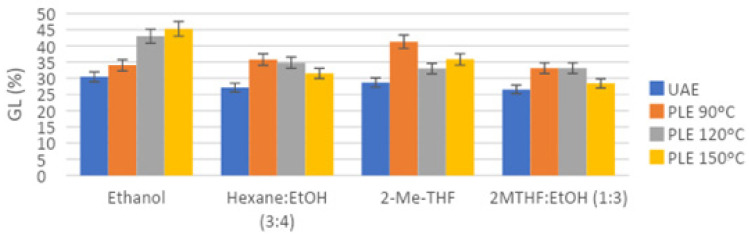
Polar lipids (GL) relative percentage in *N. gaditana* extracts, obtained by different extraction techniques, solvents, and conditions, as analyzed by HPLC-ELSD.

**Figure 6:**
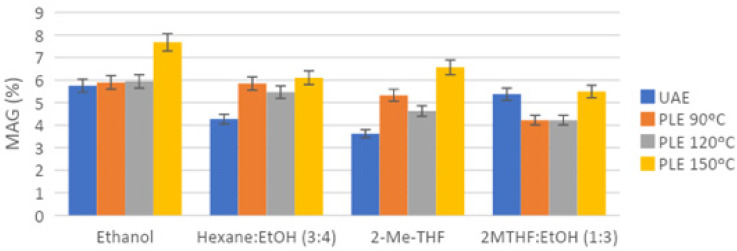
Monoacylglycerides (MAG) relative percentage in *N. gaditana* extracts, obtained by different extraction techniques, solvents, and conditions, as analyzed by HPLC-ELSD.

One of the objectives of the present study was to evaluate the composition of extracted lipids, both neutral and polar fractions. Considering the HPLC-ELSD analyses (Figure 4, Figure 5 and Figure 6), the lipid composition of *N. gaditana* was mainly formed by polar lipids. Minor percentages of TAG, FFA, 1,3-DAG, 1,2-DAG, and MAG could be observed, whereas the major peak was assigned to GL, as shown in Figure 4.

TAG content in the extracts was also analyzed (data not shown in Figures). In general, it ranged from 4.59% to 10.91%, where the highest content was found in 2-MeTHF extracts (6.72–10.91%) and in the hexane:ethanol ones (7.17–10.23%), while the lowest content was attributed to the ethanolic extracts (4.59–5.77%) as TAGs presented very different polarity to the ethanol.

**Figure 7:**
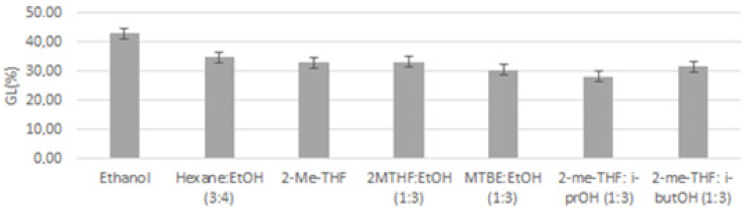
Glycolipids (GL) relative percentage in *N. gaditana* extracts obtained by PLE at 120 °C with all solvent mixtures tested, as analyzed by HPLC-ELSD.

Finally, when comparing the content of glycolipids for different pressurized extracts at the best conditions with various solvent mixtures (gathered in Figure 7 for clarity), significant differences at the 5% level were observed. Thus, PLE with ethanol at 120 °C achieved extracts with the highest content of GL (42.99%), followed by hexane:ethanol, 2-MeTHF and 2-MeTHF:ethanol extracts, which achieved an amount of GL similar between them at 5% level (32.99–34.81%). On the other hand, the solvents that achieved the lowest GL content were MTBE:ethanol, 2-MeTHF:isopropanol, and 2-MeTHF:isobutanol with a GL content that ranged between 28.10–31.51%, without significant differences at the 5% level.

### 2.4. Determination of Fatty Acid Profile of Microalgae Extracts by GC-MS

Based on the HPLC analysis and the oil yield results for each extraction, the best conditions were selected and the extracts analyzed by GC-MS to study the fatty acid profile of the samples. Comparing both extraction techniques, PLE achieved remarkably higher recovery of the biomass lipids. Due to the potential hydrolysis of compounds that may occur at the highest PLE temperatures, extracts obtained at 150 degrees were discarded, as mentioned before. Consequently, extracts obtained at 120 °C using PLE were selected for further analysis. Thus, derivatized extracts of ethanol, 2-MeTHF and their mixtures were injected in GC-MS, resulting in similar fatty acid profile, but with some significant differences in fatty acid percentage (Table 1). For instance, mixtures of 2-MeTHF and ethanol rendered higher PUFA and lower saturated fatty acid content than mixtures of MTBE:ethanol (differences observed at the 5% level). The main identified fatty acids were myristic acid (14:0), palmitic acid (16:0), palmitoleic acid (16:1), stearic acid (18:0), oleic acid (18:1), linoleic acid (18:2), γ-linolenic acid (20:3), and eicosapentaenoic acid (20:5). Two main aspects may be underlined: on the one hand, PUFAs were the main FA group (54.31–46.15%), followed by mono-unsaturated FAs (MUFAs; 30.12–24.72%); and saturated FAs (24.13–20.80%). On the other hand, within the PUFA group, n-3 PUFAs were clearly more abundant than n-6 PUFAs, resulting in a remarkable *n-6*/*n-3* ratio.

### 2.5. Application of Lipid Extraction Method to Other Microalgae

**Figure 8:**
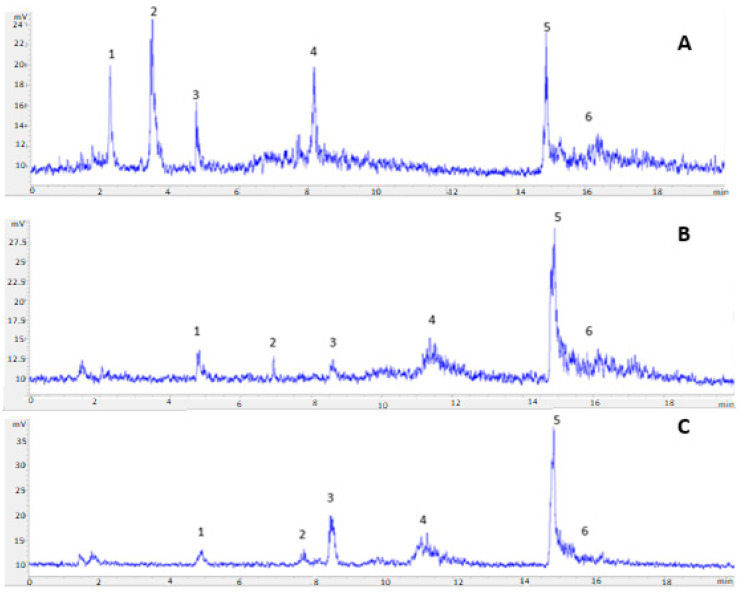
HPLC-ELSD chromatograms of *I. galbana* (**A**), *T. chuii* (**B**) *and N. gaditana* (**C**), where peak 1 corresponded to TAG, 2 to FFA, 3 to 1,3-DAG, 4 to 1,2-DAG, 5 to GL, and 6 to MAG.

The best conditions for extraction of *N. gaditana* were tested on other microalgae. Extracts obtained were analyzed using HPLC coupled with ELSD, in order to study lipid classes in each microalga (Figure 8). Peaks for FFA, TAG, DAG, GL, and MAG were identified in all cases.

GC-MS of the obtained extracts was also conducted, showing that the fatty acid profile of each microalga widely varied from each other, as expected and shown in Table 2.

*I. galbana* contained high levels of the SFA 14:0 tetradecanoic acid (22.86%) and 16:0 palmitic acid (15.11%), followed by the MUFA 16:1 palmitoleic acid (18.20%) and 18:1 oleic acid (21.12%), and the PUFAs 18:3 ALA (12.84%) and 22:6 DHA (7.27%). SFA and PUFA in *T. chuii* were found at similar levels (*p* > 0.05), where the most abundant fatty acid was 18:3 ALA (24.01%), followed by 16:0 palmitic acid (22.02%), 20:0 eicosanoic acid (20.39%), and 18:1 oleic acid (11.12%).

## 3. Discussion

Since different mixtures of hexane with ethanol or with isopropanol showed good results for extraction by UAE of lipids and other microalgae compounds such as carotenoids [19], the first purpose was to substitute hexane by a biobased solvent like 2-MeTHF. An additional goal was to achieve tunable polarity using mixtures of this renewable ether with other green solvents like alcohols, beginning with ethanol. The need of extraction solvents of intermediate polarity and mixed solvent properties has been proven in previous works that showed higher affinity for *Nannochloropsis gaditana* lipids, which are mainly polar [20,21]. As expected, the mixture of 2-MeTHF with ethanol achieved notably better results in the extraction of algal lipids than the use of 2-MeTHF alone. This result demonstrates that a more polar solvent such as ethanol combined with 2-MeTHF may be useful for polar lipid extractions. The effect may be similar to the mixture of conventional solvents with very different physicochemical properties, like chloroform and methanol, used in the traditional methods of Folch and Bligh & Dyer for the isolation of polar lipids, but here with biobased solvents instead of toxic solvents.

Second, the tested mixtures with other alcohols, isopropanol and isobutanol, produced lower oil yield, probably due to the lower polarity of these solvents compared to ethanol. Indeed, other polar alcohols such as methanol or *i*-amyl-alcohol have been recently tested in combination with other solvents, reaching interesting results with microalga biomass [13]. Thus, the combination of ethanol and 2-MeTHF using advanced pressurized solvent extraction, allows fast extraction of polar lipids from microalgae without neither toxic nor petroleum-derived solvents.

Considering the lipid profile for *N. gaditana*, it was similar to the composition found by other authors, though the relative percentages of lipid species were different. Lipid biosynthesis in microalgae follows a complex pathway, since lipids are synthesized depending on cultured conditions and, therefore, the lipid profile in *Nannochloropsis* cells could differ from one culture to another. Glycolipids (GL) are polar lipids usually accumulated in the cell membranes of the microalgae. For this reason, traditional methods such as Folch cannot efficiently release and extract glycolipids. For a more complete extraction of glycolipids, a pretreatment of the biomass is usually necessary to break the cell membrane and enhance the recovery of GL [4,8]. In our case, sonication and the combination of high pressures and temperatures seemed to be sufficient to extract GL, and results obtained with ultrasound-assisted extraction showed a recovery of GL slightly lower than PLE results using a wider range of temperatures (Figure 4). Comparing the different used extraction conditions, the analysis of extracts (Figure 4 and Figure 5) showed that the major percentage of glycolipids was found in the extracts produced by PLE with more polar solvents, especially ethanol, as expected, and at high temperatures (no significant differences at the 5% level between 150 °C and 120 °C).

The extraction temperature may affect the lipid profile, which was a determining factor for selecting the optimal temperature. One of the main observed differences was in relation to the MAG content, an indicator of possible TAG hydrolysis by different causes including high extraction temperature. MAG percentage increased significantly when PLE temperature was 150 °C (differences observed at 5% level) (Figure 5), and so, the optimal temperature was selected as 120 °C, despite a lower total lipid recovery. On the other hand, a decreasing affinity for polar lipids with increasing temperature could be observed for the 2-MeTHF extracts, as has also been described in other works [18,22,23].

At the selected temperature of 120 °C, other solvent mixtures were tested with PLE and further analyzed (Figure 7). Again, the polarity of the solvent delivered a certain selectivity, resulting in lower glycolipid content when solvents with lower polarity like MTBE and isopropanol were used. Taking into account both oil yield and glycolipid content, PLE with ethanol and PLE with 2-MeTHF:ethanol at 120 °C were selected as the optimal extracting conditions. The first one achieved higher GL content (42.01%) but lower oil yield (39.50%), whereas the second one achieved higher oil yield (46.93%) but lower GL content (33.11%), and so, both of them could be interesting for our goals.

Fatty acids identified in the oils were in accordance with results found by other authors [6,21]. As anticipated, relevant differences were observed depending on the relative percentage of glycolipids or acylglycerols in the extracts (see Table 1 for fatty acid profile of the samples). The mixtures of isopropanol, isobutanol, and MTBE produced the highest SFA content, especially with the mixture of MTBE, which means a higher affinity for non-polar compounds of this ether. On the other hand, ethanol, mixtures of 2-MeTHF:ethanol, 2-MeTHF:isobutanol, and mixtures of hexane:ethanol were the ones with higher PUFA content (36.25–38.54%) (no significant differences at 5% level), which are in agreement with the extracts with the highest glycolipid content obtained by PLE, suggesting that EPA is mainly distributed in these polar glycolipid species [6,24].

In order to test the applicability of the method for lipid extraction to other microalgae different to *Nannochloropsis gaditana*, conditions chosen as optimal in terms of oil yield, glycolipids, and EPA content were applied to another two microalgae described as important by their lipid content. *Isochrysis galbana* was chosen due to its high DHA content [25] and *Tetraselmis chuii* was chosen due to its interest in food as a lipid producer [26]. Considering the results obtained when using PLE with ethanol at 120 °C, large differences amongst the three microalgae appeared in oil yield, where *N. gaditana* was the one with the highest lipid yield (38.24%). *I. galbana* rendered lower yield (22.01%), which is in agreement with other works that found ethanol to be the optimal extracting solvent for this microalga, achieving similar results (21.69%) [25]. *T. chuii* was the one with the lowest oil yield (11.04%), which could be explained for its natural lipid content and composition and the structure of its dry biomass.

As shown in Figure 7, lipid species composition of each microalga highly varied from each other, where *I. galbana* fractions had the lower GL percentage (30.16%) of the three microalgae. Although it was an important component, this microalga was also the one with the major TAG proportion (26.09%) and FFA content (14.01%) in its extracts, which was in agreement with other works on *I. galbana*. The relatively large proportion of FFA may be due to different causes:effects of culture medium on microalgal metabolism, lipid breakdown in stressed or drying cells, and hydrolytic degradation of the biomass [19,25]. Lipid composition of *T. chuii* and *N. gaditana* analyzed by HPLC was quite similar, where glycolipids was the main peak in both microalgae, reaching a remarkably high percentage, as previously described for *N. gaditana*, with high glycolipid and omega-3 concentration [23]. Indeed, *T. chuii* showed a GL percentage (50.12%) even higher than *N. gaditana* (42.99%).

In previous works, *I. galbana* fatty acid profile was composed mainly of myristic acid (14:0) as the most abundant SFA, with important proportions of PUFAs and monounsaturated fatty acids (palmitoleic and oleic acids). ω-3 PUFAs were composed of α-linolenic acid (18:3 ω-3), stearidonic acid (18:4 ω-3), and DHA, while EPA content in other studies was very low, less than 1% of total FA. Although some authors reported a high percentage of the PUFA 18:4 n-3, it was not observed in the present study, while DHA content was variable in other articles, reporting percentages up to 10%. In general, the highest fatty acid group in *I. galbana* was of SFAs, followed by MUFAs and PUFAs, probably due to the notable DHA content [3,5,25,27,28].

EPA presence in *T. chuii* extracts was found at intermediate levels (5.13%), which is in agreement with the results obtained by other authors [25,29,30]. Furthermore, it is interesting to underline that omega-3 PUFA content was higher than omega-6, as can be seen by the *n-6*/*n-3* ratio (Table 2).

In summary, *N. gaditana* showed the highest lipid content, mainly composed by polar lipids and PUFAs, together with *I. galbana*. With regard to each PUFA, *N. gaditana* presented the highest levels of EPA, and *I. galbana* the highest levels of DHA, while for ALA, the species *T. chuii* had the highest levels, followed by *I. galbana.* Hence, the optimized proportion of 2-MeTHF and ethanol (1:3) has been applied with good results for the extraction of polar lipids with omega-3 PUFAs from several algal biomass, and it could be easily adapted to other microalgae with different lipid composition.

In this way, the results prove that the use of 2-methyl-THF could be a promising hexane substitute in solvent mixtures with alcohols when extracting by PLE more polar compounds from microalgae, promoting the principles of green chemistry through the use of alternative solvents of biological origin instead of hexane mixtures.

## 4. Materials and Methods

### 4.1. Materials

*Nannochloropsis gaditana* and *Isochrysis galbana* dry biomass was provided by AlgaEnergy S.A. (Alcobendas, Spain). *Tetraselmis chuii* was provided by Cianoalgae S.L. (Madrid, Spain).

Hexane and methanol were purchased from Lab Scan Analytical Sciences (Gliwice, Poland). Solvents (2,2,4-trimethyl pentane, methyl tert-butyl ether and 2-propanol) used for HPLC analyses were HPLC-grade and purchased from LABSCAN (Dublin, Ireland). Absolute ethanol (PRS grade), isobutanol, sodium hydrogen carbonate, and potassium hydroxide were purchased from Panreac Quimica S.A (Barcelona, Spain). 2-methyl-THF (Viridisol^®^ M) was provided by Pennakem, LLC (Memphis, TN, USA).

### 4.2. Extraction of Microalgal Biomass

**Figure 9:**
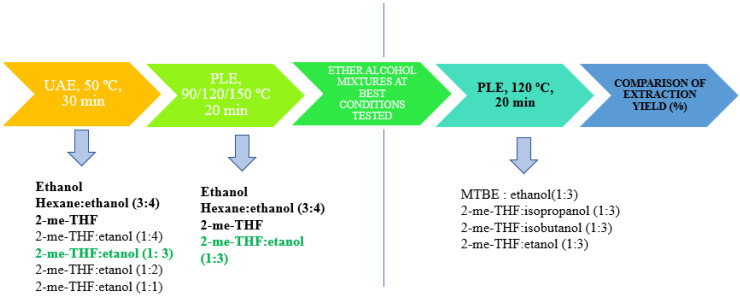
Schematic representation of the used methods of extraction.

Extractions from *Nannochloropsis gaditana* were carried out using different techniques: ultrasound assisted extraction and pressurized liquid extraction (Figure 9). The experiments were done in all cases at least in triplicate.

#### 4.2.1. Ultrasound Assisted Extraction

UAE was carried out with an ultrasound bath Elmasonic S 40H Elma brand (Singen, Germany) with automatic control of time and temperature and ultrasound frequency of 37 kHz. Samples were weighed (0.5 g of dry biomass) and the selected solvent was added in a ratio of 1:10. Extractions for each solvent were carried out at 50 °C and 30 min, based on previous works on microalgae extraction [19]. Extraction solvents used were ethanol, 2-MeTHF and mixtures of hexane:ethanol (3:4), and 2-MeTHF:ethanol (1:1, 1:2, 1:3, and 1:4).

Samples were then filtered and evaporated in a rotary evaporator (Heidolph Hei-Vap Value HB/G3, Berlin, Germany) under reduced pressure at 35 °C and dried under a nitrogen stream to constant weight. The lipid content was determined gravimetrically and was calculated as weight percentage of dry biomass. Lipid extracts obtained were stored in dark vessels under a nitrogen atmosphere at 4 °C until their analysis for less than two days.

#### 4.2.2. Pressurized Liquid Extraction

PLE was carried out with an ASE 350 DIONEX extractor (Sunnyvale, CA, USA) equipped with stainless steel extraction cells (10 mL volume). Microalgal biomass was weighed (equivalent to 1 g dry biomass), mixed with sea sand (ratio 1:10), and loaded into the extraction cell. Then, the extraction cell was filled with the different solvents used and heated to the selected temperature (90, 120, and 150 °C). The solvents used were ethanol, 2-MeTHF, and different mixtures of hexane:ethanol (3:4), 2-MeTHF:ethanol (1:3), MTBE:ethanol (1:3), 2-MeTHF:isopropyl alcohol (1:3), and 2-MeTHF:isobutanol (1:3). Static extraction time was 15 min for each experiment and the solvent volume used was 20–25 mL, depending on the temperature and pressure of each extraction. Finally, the extract was recovered using a nitrogen stream in vials of 50 mL. Samples were evaporated and treated as previously described for the UAE method.

### 4.3. Nannochloropsis Gaditana Extracts Chemical Analysis

**Figure 10:**
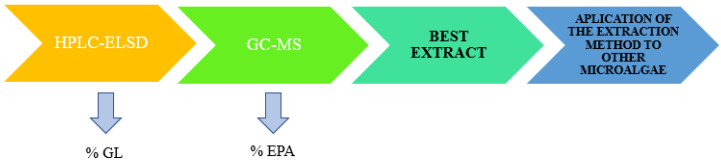
Schematic representation of the chromatographic analysis.

*Nannochloropsis* extracts were analyzed by HPLC-ELSD and GC-MS in order to determine glycolipid and EPA content, respectively, and compare different extraction conditions (Figure 10). 

#### 4.3.1. HPLC-ELSD Analysis

HPLC-ELSD analyses were performed using an Agilent 1260 Infinity HPLC equipped with an Agilent 385 (Palo Alto, CA, USA) ELSD and DAD instruments. The chromatographic separation of the different species of lipids (neutral and polar lipids) [19] was carried out with a silica normal-phase ACE (250 mm × 4.6 mm i.d., 5 μm) column maintained at 30 °C using a ternary gradient as follows: 0–2 min, 99.5% A and 0.5% B; at t = 6.5 min, 70% A and 30% B; at t = 11 min, 63% A, 27% B and 10% C; at t = 18 min, 99.5% A and 0.5% B; and at t = 20 min, 99.5% A and 0.5% B. Eluent A consisted of 2,2,4-trimethylpentane, eluent B consisted of methyl tert-butyl ether, and eluent C consisted of 2-propanol. The flow rate was 2.0 mL/min except for minutes 13 to 16, which was 1.0 mL/min. Optimal signal and resolution were attained with the following ELSD conditions: evaporator temperature = 30 °C; nebulizer temperature = 30 °C; and evaporator gas N2 = 1.6 SLM.

Lipid species were identified using commercial standards for neutral lipids like TAG, DAG, MAG, and FFA. Glycolipids were identified using a standard isolated by the research group from spinach leaves, as previously described [4,6,19]. Results were expressed as the individual relative percentage of each lipid species present in the sample (normalized areas).

#### 4.3.2. Fatty Acid Composition by GC-MS

Fatty acid methyl esters standard (Supelco 37 FAME Mix) was from Supelco (Bellefonte, PA, USA).

Fatty acid composition of the obtained extracts was analyzed by GC–MS. Previous to analysis on an Agilent GC-MS series 5975 MSD (Palo Alto, California., USA), fatty acid methyl esters (FAMEs) were freshly prepared by base-catalyzed methanolysis of the glycerides (KOH in methanol). FAMEs were separated using a HP 88 capillary column (100 m × 0.25 mm, i.d. 0.2 μm) (Agilent, Santa Clara, California, USA). One μL sample was injected using a split ratio of 1:100. The column was held at 175 °C for 10 min after injection, the temperature programmed at 3 °C/min to 220 °C, and maintained for 20 more minutes. Helium was used as a carrier gas, at a constant column flow rate of 1.5 mL/min. Injector temperature was 250 °C and the detector temperature was 230 °C. The mass spectrometer was operated at 70 eV with a mass range from 30 to 400 amu. Fatty acids methyl esters were identified by comparing their retention times and mass spectra (NIST MassSpectral Library Version 2.0) with those obtained from the standards. Results were expressed as the individual relative percentage of each fatty acid over all FAMEs in each sample.

### 4.4. Statistical Analyses

Statistical analysis was performed using the SISA (Simple Interactive Statistical Analysis) online software [31]. The results were expressed as the mean of the experiments including its standard deviation. The data were subjected to the t-test to examine whether the means of two groups differed from one another. To compare the means of three or more independent samples (treatments) simultaneously, the data were subjected to a one-way analysis of variance (ANOVA) using the F test for discrimination between means (*p* < 0.05) including Tukey’s HSD (honest significant difference) test as a single-step multiple comparison procedure to find means that were significantly different from each other.

## 5. Conclusions

The studied combinations of biobased solvents provided green alternatives for microalgal lipid extraction in short times using advanced techniques like PLE and UAE. Specifically, optimized extraction of *Nannochloropsis gaditana* biomass proved the efficacy of renewable solvents mixtures like 2-methyl-THF:ethanol to efficiently extract polar lipids with omega-3 PUFAs from several microalgal biomass. Solvent properties were modified and adjusted by mixing green solvents in different proportions, improving the oil extraction yield and lipid fractionation with mixtures of different polarity like ethers and several alcohols. The optimized proportion of biobased solvents of 2-methyl-THF:ethanol (1:3 *v/v*) resulted in the highest algal oil yields either using PLE or UAE, whereas extraction with only 2-methyl-THF was not optimal for polar lipid production.

Analysis of lipid classes of the samples by HPLC-ELSD and fatty acid profiles by GC-MS showed a different fractionation of algal lipids in the produced extracts. Moreover, ethanolic PLE extracts of *N. gaditana* at 120 °C were the ones that contained higher amounts of glycolipids and EPA due to the higher polarity of the solvent and method efficiency.

The optimized method was applied to other microalgae, *Isochrysis galbana* and *Tetraselmis chuii,* proving the applicability for fast lipid extraction. *N. gaditana* showed the highest lipid content, mainly composed by polar lipids and PUFAs. With regard to omega-3 PUFAs, *N. gaditana* presented the highest levels of EPA, and *I. galbana* the highest levels of DHA, while *T. chuii* had the highest levels of ALA, followed by *I. galbana*.

In summary, biobased solvents tested in UAE and PLE demonstrate that both techniques are useful for microalgae lipid extraction from different algal species, with PLE reaching different lipid fractionation and higher oil yields. Consequently, the results show the potential of renewable solvents mixtures like 2-methyl-THF:ethanol to efficiently extract lipids from microalgal biomass by PLE. Since the use of alternative solvents of biological origin instead of hexane mixtures promotes the principles of green chemistry, the results prove that the use of 2-methyl-THF could be a promising substitute for hexane or chloroform in solvent mixtures with alcohols when extracting more polar compounds from microalgae.

## Figures and Tables

**Table 1:** Fatty acid composition (as percentage of total fatty acids) determined by GC-MS of the best extracts obtained from *Nannochloropsis gaditana* using PLE at 120 °C and different extraction solvents.

Fatty Acid	Rt (min)	% Fatty Acids						
		Ethanol	2-MeTHF	2-MeTHF:Ethanol (1:3)	Hexane: Ethanol (3:4)	MTBE:Ethanol (1:3)	2-MeTHF:isobutanol (1:3)	2-MeTHF:isopropanol (1:3)
*14:0*	9.70	4.74 ± 0.11 ᵃ	5.34 ± 0.35 ᵃ	4.76 ± 0.76 ᵃ	4.73 ± 0.28 ᵃ	5.18 ± 0.88 ᵃ	4.70 ± 0.25 ᵃ	4.77 ± 0.46 ᵃ
*16:0*	11.77	16.64 ± 1.21 ᵃ	17.11 ± 0.66 ᵃ	16.83 ± 0.93 ᵃ	14.47 ± 0.77 ᵃ	18.19 ± 0.50 ᵃ	17.13 ± 1.09 ᵃ	17.65 ± 0.57 ᵃ
*16:1*	12.50	23.07 ± 0.74 ᵃ	25.13 ± 1.01 ᵃ	24.57 ± 0.48 ᵃ	23.44± 0.62 ᵃ	25.74 ± 1.36 ᵃ	24.96 ± 0.67 ᵃ	25.31 ± 0.18 ᵃ
*18:0*	14.59	0.49 ± 0.23 ᵃ	0.40 ± 0.10 ᵃ	0.61 ± 0.55 ᵃ	0.97± 0.15 ᵃ	0.76 ± 0.20 ᵃ	0.46± 0.43 ᵃ	0.39 ± 0.13 ᵃ
*18:1*	15.59	4.17 ± 0.12 ᵃ	4.54 ± 0.50 ᵃ	4.47 ± 0.58 ᵃ	4.42± 0.59 ᵃ	4.38 ± 0.55 ᵃ	4.34 ± 0.82 ᵃ	4.49 ± 0.28 ᵃ
*18:2*	17.12	3.37 ± 1.01 ᵃ	4.14 ± 0.34 ᵃ	3.74 ± 0.26 ᵃ	3.75± 0.28 ᵃ	3.57 ± 0.64 ᵃ	3.69 ± 0.72 ᵃ	3.29 ± 1.09 ᵃ
*20:3*	21.90	0.51 ± 0.52 ᵃ	0.60 ± 0.12 ᵃ	0.79 ± 0.33 ᵃ	1.19 ± 0.40 ᵃ	0.41 ± 0.16 ᵃ	0.62 ± 0.41 ᵃ	0.65 ± 0.10 ᵃ
*20:4*	22.69	7.92 ± 0.48 ᵃ	7.98 ± 0.58 ᵃ	8.60± 0.85 ᵃ	8.46 ± 0.94 ᵃ	8.26 ± 0.88 ᵃ	7.77± 0.85 ᵃ	8.01 ± 1.05 ᵃ
*20:5*	24.61	37.38 ± 0.66 ᵃᵇ	34.93 ± 0.69 ᵇ	36.25 ± 0.42 ᵃᵇ	38.54 ± 0.93 ᵃ	33.91 ± 0.55 ᵇ	36.33 ± 0.68 ᵃᵇ	35.42 ± 1.33 ᵃᵇ
*SFA*		21.87 ᵃ	22.85 ᵇ	22.20 ᵃᵇ	20.17 ᶜ	24.13 ͩ	22.29 ᵃᵇ	22.81 ᵇ
*MUFA*		27.24 ᵃ	29.67 ᵇ	29.04 ᵇ	27.86 ᵃ	30.12 ᵇ	29.30 ᵇ	29.80 ᵇ
*PUFA*		49.18 ᵃ	47.65 ᵇ	48.74 ᵃ	51.94 ᵃ	46.15 ᵇ	48.41 ᵃ	47.37 ᵇ
*n-3*		37.89 ᵃ	35.54 ᵇ	37.04 ᵃ	39.73 ᶜ	34.32 ᵇ	36.95 ᵃᵇ	36.07 ᵇ
*n-6*		11.29 ᵃ	12.12 ᵃ	12.34 ᵃ	12.21 ᵃ	11.83 ᵃ	11.46 ᵃ	11.30 ᵃ
*n-6/n-3*		0.30 ᵃ	0.34 ᵃ	0.33 ᵃ	0.31 ᵃ	0.34 ᵃ	0.31 ᵃ	0.31 ᵃ

Rt, retention time; SFA, saturated fatty acids; MUFA, monounsaturated fatty acids; PUFA, polyunsaturated fatty acids; Results expressed as percentage over the total content (relative content). Values are the mean ± SD of three determinations; a–d: Different lowercase letters denote significant differences between samples from the same row (*p* < 0.05).

**Table 2:** Fatty acid composition (as percentage of total fatty acids) determined by GC-MS of the extracts obtained from *Nannochloropsis gaditana*, *Isochrysis galbana*, and *Tetraselmis chuii* using PLE at 120 °C with ethanol as the extraction solvent.

Fatty Acid	% Fatty Acids		
	*N. gaditana*	*I. galbana*	*T. chuii*
*14:0*	4.74 ± 0.11 ᵃ	22.86 ± 0.59 ᵇ	-
*16:0*	16.64 ± 1.21 ᵃ	15.11 ± 0.87 ᵃ	22.02 ± 0.55 ᵃ
*16:1*	23.07 ± 0.74 ᵃ	18.2 ± 0.33 ᵃ	1.45 ± 0.57 ᵇ
*17:0*	-	2.85 ± 0.15 ᵃ	-
*18:0*	0.49 ± 0.23 ᵃ	1.60 ± 0.28 ᵇ	-
*18:1*	4.17 ± 0.12 ᵃ	9.93 ± 0.46 ᵇ	11.12 ± 0.22 ᵇ
*18:2*	3.37 ± 1.01 ᵃ	9.33 ± 0.13 ᵇ	-
*18:3*	-	12.84 ± 0.40 ᵃ	24.01 ± 0.72 ᵇ
*20:0*	-	-	20.39 ± 0.86 ᵃ
*20:3*	0.51 ± 0.52 ᵃ	-	7.39 ± 0.28 ᵇ
*20:4*	7.92 ± 0.48 ᵃ	-	-
*20:5*	37.38 ± 0.66 ᵃ	-	5.13 ± 0.74 ᵇ
*22:0*	-	-	3.56 ± 0.33 ᵃ
*22:6*	-	7.27 ± 0.28 ᵃ	-
*SFA*	21.87 ᵃ	42.42 ᵇ	42.41 ᵇ
*MUFA*	27.24 ᵃ	28.13 ᵇ	12.57 ᶜ
*PUFA*	49.18 ᵃ	29.44 ᵇ	40.09 ᶜ
*n-3*	37.89 ᵃ	20.11 ᵇ	29.14 ᶜ
*n-6*	11.29 ᵃ	9.33 ᵇ	7.39 ᶜ
*n-6/n-3*	0.30 ᵃ	0.46 ᵃ	0.25 ᵃ

Rt, retention time; SFA, saturated fatty acids; MUFA, monounsaturated fatty acids; PUFA, polyunsaturated fatty acids. Results expressed as percentage over the total content (relative content). Values are the mean ± SD of three determinations; a–c: Different lowercase letters denote significant differences between samples from the same row (*p* < 0.05).

## Data Availability

The data presented in this study are available on request from the corresponding author.
